# Medical Society of Georgia

**Published:** 1859-02

**Authors:** 


### EDITORIAL AND MISCELLANEOUS.

### MEDICAL SOCIETY OF GEORGIA.

Feeling a deep interest in the welfare of this body, over which the Senior Editor of this Journal was called to preside for the present year, we would desire to direct the attention of the profession throughout the State to the fact, that it holds its next Annual Meeting in the City of Atlanta, on the second Wednesday in April, 1859.

It would be a work of supererogation to go into an argument to prove the advantages of Medical Associations, and this is not our object, but we feel that something ought to be done to arouse the Medical men of the State from their apparent forgetfulness of the great interests which they have involved in the question, whether the State Medical Society shall be the instrument of the incalculable amount of good, it may be made to accomplish?

We shall not hesitate to say, that in our judgment, the permanent prosperity of this body far exceeds in value that of the American Medical Association, to the Medical men of the State of Georgia.

We regard it as the only *real bond of Union* in the shape of organization, to the permanency of which, we can look with any confidence, and besides, as the only rightful guardian of the interests of the profession, however heterodox the doctrine may be, to those who look beyond its jurisdiction, for all the power to regulate Medical matters within our borders.

We know of no "higher law" in Medicine, than that which emanates from the assembled wisdom and authority, of the large number of Medical men to be found throughout the length and breadth of this broad *Sovereignty.*

Entertaining such views then, we would desire to urge in the most earnest manner, our Medical brethren from every portion of the State to come up in full representation to the Annual Meeting of their Society, assuring them in the name of the Profession of this city, that they will meet with a most cordial welcome.

Being placed, as we are, at the most accessible point to every portion of the State, we confidently anticipate the largest meeting since the organization of the Society, and the inauguration of a new and brighter era in its history.

In conclusion, we desire to commend the subject to the attention of the various Medical Journals of the State, with the request that they will unite with us, in the effort to bring up a large representation to the next meeting of the Society.

As there may be some misapprehension upon the part of those who have not heretofore attended the deliberations of this body, in reference to the terms of admission, we would add, that any true Medical man in regular standing, is eligible to membership—without a fee—either upon presenting himself, or being vouched for by some member of the Society.

